# Interleukin‐22 promotes development of malignant lesions in a mouse model of spontaneous breast cancer

**DOI:** 10.1002/1878-0261.12598

**Published:** 2019-12-04

**Authors:** Gajendra K. Katara, Arpita Kulshrestha, Sylvia Schneiderman, Valerie Riehl, Safaa Ibrahim, Kenneth D. Beaman

**Affiliations:** ^1^ Center for Cancer Cell Biology, Immunology and Infection Chicago Medical School Rosalind Franklin University of Medicine and Science North Chicago IL USA; ^2^ Department of Microbiology and Immunology Faculty of Pharmacy Cairo University Egypt

**Keywords:** breast cancer, EMT, IL‐22, invasion, malignancy, metastasis

## Abstract

Interleukin (IL)‐22 is recognized as a tumor‐supporting cytokine and is implicated in the proliferation of multiple epithelial cancers. In breast cancer, the current knowledge of IL‐22 function is based on cell line models and little is known about how IL‐22 affects the tumor initiation, proliferation, invasion, and metastasis in the *in vivo* system. Here, we investigated the tumor stage‐specific function of IL‐22 in disease development by evaluating the stage‐by‐stage progression of breast cancer in an IL‐22 knockout spontaneous breast cancer mouse model. We found that among all the stages, IL‐22 is specifically upregulated in tumor microenvironment (TME) during the malignant transformation stage of breast tumor progression. The deletion of IL‐22 gene leads to the arrest of malignant transition stage, and reduced invasion and tumor burden. Administration of recombinant IL‐22 in the TME does not influence *in vivo* tumor initiation and proliferation but only promotes malignant transformation of cancer cells. Mechanistically, deletion of IL‐22 gene causes downregulation of epithelial‐to‐mesenchymal transition (EMT)‐associated transcription factors in breast tumors, suggesting EMT as the mechanism of regulation of malignancy by IL‐22. Clinically, in human breast tumor tissues, increased number of IL‐22^+^ cells in the TME is associated with an aggressive phenotype of breast cancer. For the first time, this study provides an insight into the tumor stage‐specific function of IL‐22 in breast tumorigenesis.

## Graphical abstract

This study identifies tumor stage‐specific function of interleukin (IL)‐22 in breast cancer. IL‐22 inactivation causes downregulation of epithelial‐to‐mesenchymal transition genes in tumor cells. This leads to inhibition in the malignant transition stage of tumor progression and reduced invasion and metastasis. In human breast tumor tissues, increased number of IL‐22^+^ cells in tumor microenvironment is associated with an aggressive phenotype of breast cancer.

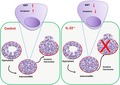

**Abbreviations**
DCIS: ductal carcinoma *in situ*
EMT: epithelial‐to‐mesenchymal transition
IHC: immunohistochemistry
IL‐10R2: interleukin‐10 receptor‐2
IL‐22: interleukin‐22
IL‐22^−/−^: IL‐22 knockout mouse
IL‐22^+/+^: IL‐22 wild‐type mouse
IL‐22BP: interleukin‐22 binding protein
IL‐22RA1: interleukin‐22 receptor‐1
LNM: lymph node metastasis
MIN: mammary intraepithelial neoplasia
MMP: matrix metalloproteinase
MMTV: mouse mammary tumor virus
PyMT: polyomavirus middle T antigen
qPCR: quantitative real‐time PCR
STAT: signal transducer and activator of transcription
Th1: T‐helper cell type‐1
Th17: T‐helper cell type‐17
Th22: T‐helper cell type‐22
TMA: tissue microarray
TME: tumor microenvironment
WB: western blot

## 1 Introduction

Breast cancer is the most common malignancy in women (Bray *et al.*, 2018). The majority of breast cancer‐associated deaths (90%) are caused by metastases of cancer cells rather than primary tumors (Weigelt *et al.*, 2005). Metastasis is a complex and multistep process that involves detachment of specialized cancer cells from the primary tumor, invasion of surrounding stroma, entry into the circulation, and colonization in distant organs. Cancer cells within primary tumors, with genetic alterations, are considered responsible for metastasis. These cancer cells attain mesenchymal characteristics and become malignant/invasive. The local microenvironment or stroma around primary tumors plays an important role in influencing the invasive behavior in these cancer cells. This stroma contains immune cells, cytokines, growth factors, fibroblasts, endothelial cells, and extracellular matrix (Quail and Joyce, 2013). Unlike cancer cells, stromal cell types and the secreted cytokines within the tumor microenvironment (TME) are genetically stable and thus represent an attractive therapeutic target with reduced risk of resistance and tumor recurrence.

Interleukin‐22 (IL‐22) is a cytokine of the IL‐10 family and the only known cytokine that is produced by immune cells and acts primarily on the cells of nonhematopoietic origin such as epithelial cells, fibroblasts, renal tubular cells, keratinocytes, trophoblasts, and pancreatic ductal cells expressing the receptor [IL‐22 receptor‐1 (IL‐22RA1)]. The binding of IL‐22 to IL‐22RA1 triggers phosphorylation of signal transducer and activator of transcription 3 (STAT3) and induction of various signaling pathways, which include JAK/STAT, mTOR, AKT, and MAPKs (Sabat *et al.*, 2014). IL‐22 binding protein (IL‐22BP) is a soluble receptor of IL‐22 and exhibits higher affinity to IL‐22 than IL‐22RA1. The binding of IL‐22 to IL‐22BP blocks IL‐22RA1 activation and inhibits the downstream signaling. IL‐22BP is produced by dendritic cells and normal tissues from spleen, stomach, intestines, lungs, skin, placenta, and breast. Tissue injury and bacterial infections cause release of IL‐22BP as a mechanism to suppress IL‐22‐mediated inflammation (Sabat *et al.*, 2014). IL‐22 has been implicated in various pathological conditions including autoimmune, inflammatory, and infectious diseases and cancer (Lim and Savan, 2014). In cancer, the role of IL‐22/IL‐22RA1 axis is complex and context‐dependent. Elevated levels of IL‐22 are reported in cancer patients and associated with poor survival rate (Rui *et al.*, 2017; Voigt *et al.*, 2017). IL‐22 stimulates STAT‐3‐mediated cell proliferation and stemness in cancer cells in colon, liver, gastric, pancreatic, and lung cancers (Lim and Savan, 2014). However, anticancer effects of IL‐22 are also reported in cancer where it attenuates the growth of cancer cells through G2/M cell cycle arrest leading to reduced cell proliferation and tumor weight (Zhang *et al.*, 2011). High expression of IL‐22RA1 in cancer cells is associated with poor prognosis in pancreatic ductal carcinoma (DC) patients (He *et al.*, 2018).

In breast cancer, relatively few studies have been performed to understand the role of IL‐22 in disease pathogenesis. The current knowledge of IL‐22 function is based on advanced‐stage cancer cell line models where it has been shown that IL‐22 stimulates cell proliferation, transformation, and migration in human/mouse cancer cell lines (Kim *et al.*, 2014; Rui *et al.*, 2017; Wang *et al.*, 2018). Voigt *et al*. have recently demonstrated that inhibition of IL‐22 production through IL‐1R antagonist anakinra can reduce tumor burden in a transplant tumor model and identified Th22 cells as a primary source of IL‐22 in breast cancer. Due to numerous limitations with cell line models, understanding the role of IL‐22 in disease pathogenies is still incomplete. The absence of complete *in vivo* studies also contributes to limited understanding of IL‐22 function in disease pathogenesis.

Here, using an IL‐22 knockout breast cancer mouse model, we have explored the cancer cell malignancy‐associated role of IL‐22 in breast cancer pathogenesis. We show that IL‐22 is highly expressed in the TME during the invasion stage of breast tumor progression and inactivation of IL‐22 gene leads to the inhibition in the malignant transition stage and reduced tumor growth. In human breast tumors, the number of IL‐22^+^ cells positively correlates with the aggressive phenotype of breast cancer.

## 2 Materials and methods

### 2.1 Generation of IL‐22^−/−^/PyMT mice

Interleukin‐22 knockout (IL‐22^−/−^) mice were laboratory‐generated as described before (Dambaeva *et al.*, 2018). For generating an IL‐22 knockout spontaneous breast cancer mouse model, IL‐22 wild‐type (IL‐22^+/+^) or IL‐22^−/−^ mice (C57/B6) were crossed with mouse mammary tumor virus‐polyomavirus middle T antigen (MMTV‐PyMT) transgenic mice (FVB; Jackson Laboratories, Bar Harbor, ME, USA). The MMTV‐PyMT transgenic mice carry PyMT oncogene under the control of regulatory promoter for the MMTV long terminal repeat, which is specifically expressed in mammary epithelium. The IL‐22^+/+^ or IL‐22^−/−^ mice were bred seven generations into the FVB genetic background. Female IL‐22^+/+^ or IL‐22^−/−^ mice (FVB) were crossed with male IL‐22^+/+^ or IL‐22^−/−^ MMTV‐PyMT transgenic mice (FVB) to generate IL‐22^+/+^/PyMT or IL‐22^+/−^/PyMT or IL‐22^−/−^/PyMT mice (FVB). Female mice of the same age‐group of IL‐22^+/+^/PyMT or IL‐22^+/−^/PyMT or IL‐22^−/−^/PyMT genotype were used for experiments. Male IL‐22^+/+^/PyMT or IL‐22^−/−^/PyMT genotype and PyMT noncarrier female mice of IL‐22^+/+^ or IL‐22^−/−^ genotype were used for breeding purposes. IL‐22^+/+^/PyMT mice were used as controls for H&E and Ki‐67 staining. For * in vivo* cancer growth assay, PBS‐injected wild‐type IL‐22^+/+^/PyMT mice were used as control mice, whereas recIL‐22‐injected wild‐type IL‐22^+/+^/PyMT mice were used as test mice. For *in vitro* cell proliferation, migration, and invasion assays; tumor cells isolated from IL‐22^+/+^/PyMT mice and stimulated with PBS were used as controls. For *in vivo* IL‐22 supplementation experiment, PBS‐injected knockout IL‐22^−/−^/PyMT mice were used as control mice, whereas recIL‐22‐injected IL‐22^−/−^/PyMT mice were used as test mice. All the animal experiments were performed in accordance with the Institutional Animal Care and Use Committee of the Rosalind Franklin University of Medicine and Science, North Chicago, Illinois.

### 2.2 Antibodies and reagents

Mouse monoclonal anti‐Ki‐67 (Abcam, Cambridge, UK, Cat. No. 15580), rabbit anti‐laminin‐1α (Abcam, Cat. No. 11575), rabbit anti‐IL‐22 (Abcam, Cat. No. 18499), anti‐matrix metalloproteinase (MMP)‐3 (Invitrogen, Carlsbad, CA, USA, Cat. No. MA5‐17123), GAPDH (Cell Signaling, Danvers, MA, USA, Cat. No. 2118s), anti‐CD326‐APC (Biolegend, San Diego, CA, USA, Cat. No. 118214), anti‐CD45‐BV421 (Biolegend, Cat. No. 103134), and rabbit and mouse IgG isotype controls (Sigma, St. Louis, MO, USA). EnVision + Dual Link System‐HRP polymer was purchased from Agilent (Santa Clara, CA, USA). Permount from Fisher Scientific (Hampton, NH, USA) was used as a mounting medium. Carmine Alum was purchased from StemCell Technologies (Vancouver, Canada).

### 2.3 Whole‐mount mammary gland carmine staining

Inguinal mammary glands were collected from females in the following stages of breast cancer progression: initiation (4 weeks old), hyperplasia (6 weeks old), adenoma (8 weeks old), early carcinoma (10 weeks old), and late carcinoma (12–14 weeks old). Mammary whole‐mount analysis was performed as described previously (Plante *et al.*, 2011). Glands were dissected and spread out on a glass slide, soaked overnight in Carnoy’s fixative, washed with 70% ethanol and gradually rehydrated with water, and stained with Carmine Alum. Stained glands were cleared in ethanol and xylene and mounted with Permount. Pictures were taken with a MZ75 light microscope (Leica, Wetzlar, Germany). Ductal elongation and bifurcation was quantified as reported previously (Alston‐Mills *et al.*, 2011). Quantitative image analysis of the mammary gland whole mounts was performed by using the aperio imagescope software (Leica Biosystems, Wetzlar, Germany).

### 2.4 Histology and immunohistochemistry

Tissue sections of 5 μm size from the paraffin‐embedded breast tumors and lung tissue were used. For histology, sections were deparaffinized in xylene and alcohol and stained with Mayer’s hematoxylin and 0.1% eosin. The immunohistochemistry (IHC) was performed using Dako EnVision + HRP‐DAB system in accordance with the manufacturer’s instructions. Briefly, fixed frozen sections were boiled in sodium citrate buffer (pH = 6.0) for antigen retrieval. These sections were blocked for endogenous peroxidase activity by using dual‐peroxidase block and for protein blocking by using 5% BSA. Tissue sections were incubated with primary antibodies overnight at 4 °C followed by washing with PBST and incubation with secondary antibody polymer for 20 min at room temperature. DAB was used as a chromogen to detect specific proteins in tissue sections. The sections were counterstained with Mayer’s hematoxylin and mounted in Permount mounting medium. Tissue sections were visualized and pictures were taken in a Leica ICC 50W light microscope (Leica Biosystems).

### 2.5 Isolation of PyMT tumor cells

Mammary tumors dissected from female mice were minced and dissociated enzymatically at 37 °C for 2 h in culture medium [Dulbecco’s modified Eagle’s medium (DMEM)/F12] and 2% FBS supplemented with 300 U·mL^−1^ collagenase and 100 U·mL^−1^ hyaluronidase (StemCell Technologies). The resulting cell suspension was suspended in 0.25% trypsin‐EDTA for 2 min. After collection, the pellets were digested at 37 °C for 1 h in culture medium supplemented with 2 mg·mL^−1^ dispase and 0.1 mg·mL^−1^ DNase (StemCell Technologies). Dissociated cells were then depleted of red blood cells by suspending in RBC lysis buffer for 3 min and finally filtered through a 40‐mm mesh. The filtered cell suspensions were further used for tumor cell purification using a mouse tumor cell isolation kit (Miltenyi Biotec, Bergisch Gladbach, Germany, Cat. No. 130‐110‐187) as per the manufacturer’s instructions. The identity of tumor cells was ascertained by staining of tumor cells with anti‐CD326 (EpCAM) and CD45 lineage marker in flow cytometry. Samples showing > 90% CD326+ cells in flow‐through were used for further experiments. The representative flow cytometry results are shown in Fig. S1. These tumor cells were cultured in DMEM high‐glucose medium and used for cell proliferation, migration, and invasion assays.

### 2.6 Cell proliferation, migration, and invasion assays

The cell proliferation assay was performed using Promega's CellTiter Cell Viability Assay (Promega, Madison, WI, USA) according to the manufacturer's protocol. Briefly, purified PyMT cells or MDA human mammary breast epithelial carcinoma cell line (ATCC, City of Manassas, VA, USA) were plated into 96‐well plates (500 cells·well^−1^) in DMEM and stimulated with PBS or recIL‐22 (20 ng·mL^−1^) and incubated at 37 °C in a 5% CO_2_ atmosphere. At 48 h, cell proliferation reagent was added to each well and incubated for 1 h. The cell number was measured through absorbance by a 96‐well microplate reader. A wound‐healing assay was used to demonstrate the migration potential of PyMT cells treated with PBS or recIL‐22 as described before (Katara *et al.*, 2014). PyMT cells were subjected to *in vitro* scratch assay and images captured at 0, 24, and 48 h after incubation using a phase‐contrast microscope. The invasion assay was conducted using a CytoSelect 24‐well cell invasion assay kit (Cell Biolabs Inc., San Diego, CA, USA). Briefly, PyMT cells (1 × 10^5^) were suspended in 200 µL of serum‐free DMEM, stimulated with PBS or recIL‐22 (20 ng·mL^−1^), and added to the upper inserts. DMEM (500 µL) with 10% FBS was added to the lower chamber. After 48 h, invaded cells in the lower chamber were used for fluorometric analysis as per the manufacturer’s instructions.

### 2.7 IL‐22 bioassay

The amount of IL‐22 in breast tumors was analyzed by Milliplex MAP kit (Millipore) in total protein lysates prepared from primary tumors from 4‐ to 14‐week‐old IL‐22^+/+^/PyMT or IL‐22^−/−^/PyMT mice. Protein lysates were prepared using a total protein extraction kit (Millipore, Burlington, MA, USA) as per the manufacturer’s instructions. Equal amounts of tumor tissues were used for the assay.

### 2.8 RNA preparation and real‐time PCR

Total RNA was isolated from tumors using RNeasy Mini Kit (Qiagen, Hilden, Germany), and single‐stranded cDNA was synthesized using High‐Capacity cDNA Synthesis Kit (Invitrogen). Quantitative real‐time PCR (qPCR) was performed using cDNA‐specific FAM‐MGB‐labeled TaqMan primer sets (Invitrogen) for IL‐22RA, IL‐22BP, Snail‐1, Snail‐2, Twist‐1, Zeb‐1, and MMP‐3 genes. VIC‐MGB‐labeled *GAPDH* was used as an endogenous control.

### 2.9 Tissue microarray

To investigate the clinical significance of IL‐22 expression in breast cancer, IL‐22 staining was performed by IHC. Tissue microarray (TMA) consisting of primary tumor tissue sections from DC *in situ* (DCIS), grade 1–3 invasive DC, metastatic‐to‐lymph node DC (LNM‐DC), invasive lobular carcinoma, and inflammatory breast cancer was procured from the Cooperative Human Tissue Network, University of Virginia. This TMA contains seven cases of cancer tissues of each grade along with matched normal breast tissue sections. The institutional IRB committee was consulted before using these arrays. Due to anonymized or de‐identified specimen’s nature of these tissue arrays, and because the study does not include any access to identifiable private information, IRB review and approval was not required for the study. IHC was performed as described above using anti‐IL‐22 antibody (Abcam). The sensitivity of this antibody was tested by IHC in spleen and normal mammary gland (data not shown), and the specificity was tested by western blot (WB; Fig. S4).

### 2.10 Immunoblotting

Total protein lysates were mixed with sample buffer and heated at 95 °C for 5 min. Equal amounts of protein were subjected to SDS/PAGE and transferred to nitrocellulose. The membranes were blocked in protein‐free PBS blocking buffer (Thermo Scientific) for 1 h at room temperature. Primary and secondary Ab incubations were performed in protein‐free blocking buffer/0.05% Tween‐20 for 1 h at room temperature. For all membranes, protein signals were detected using an Odyssey imaging instrument and analyzed using instrument software (Li‐Cor Biosciences, Lincoln, NE, USA).

## 3 Results

### 3.1 Disruption of IL‐22 gene impairs mammary tumor development in the MMTV‐PyMT mouse tumor model

**Figure 1:**
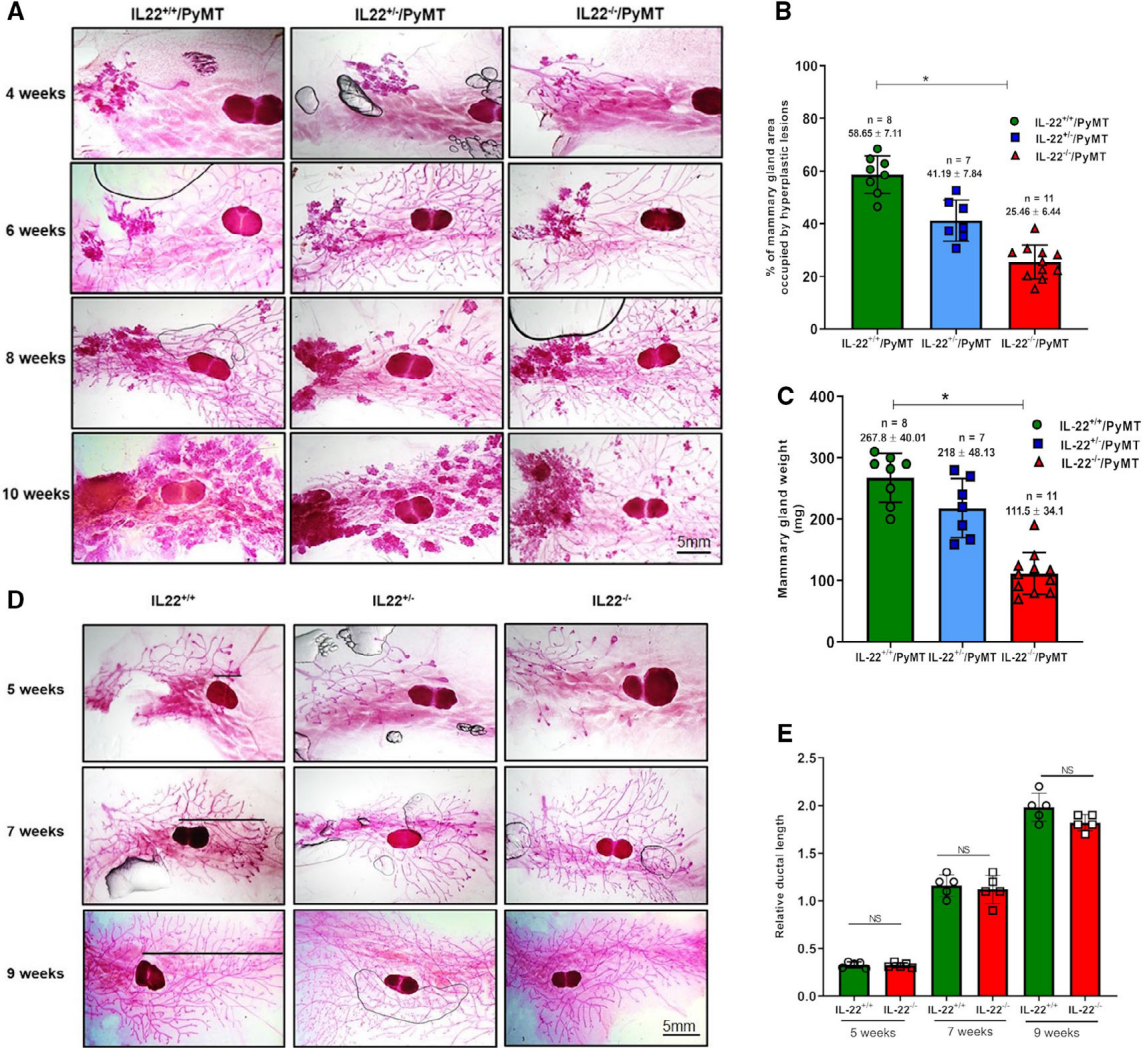
IL‐22 knockout (IL‐22^−/−^/PyMT) mice display reduced hyperplastic lesion formation. (A) Representative mammary gland whole mounts from 4‐ to 10‐week‐old virgin IL‐22^+/+^or IL‐22^+/−^ or IL‐22^−/−^PyMT mice. Four‐week‐old mice (*n* = 7 per group), 6‐week‐old mice (*n* = 7 for IL‐22^+/+^ and IL‐22^+/−^PyMT and *n* = 9 for IL‐22^−/−^PyMT), 8‐week‐old mice (*n* = 10 per group), and 10‐week‐old mice (*n* = 8 for IL‐22^+/+^PyMT, *n* = 7 for IL‐22^+/−^ PyMT and *n* = 11 for IL‐22^−/−^PyMT mice (scale bar, 5 mm). (B) Quantification of the area occupied by hyperplastic lesions expressed as a percentage (± SD) of the total mammary gland surface. The number of mice used in the experiment is indicated in the figure. Age: 10 weeks. *P < 0.01, Student’s *t*‐test. (C) Quantification of average mass (± SD) of inguinal mammary glands. The number of mice used in the experiment is indicated in the figure. Age: 10 weeks. *P < 0.01, Student’s *t*‐test. (D) Carmine Alum staining of mammary gland whole mounts from 5‐ to 9‐week‐old virgin non‐PyMT female IL‐22^+/+^or IL‐22^+/−^ or IL‐22^−/−^ mice. Lines represent ductal migration from the beginning of LN to the tip of furthest migrating duct. *n* = 5 for each time point and each group (scale bar, 5 mm). (E) Relative ductal length was measured as a difference between whole length of the gland (from nipple end to tip of ductal tree) and ductal migration (from the beginning of LN to the tip of ductal tree). Data represent mean ± SD, *n* = 5. NS, nonsignificant.

To extend our knowledge of the function of IL‐22 in breast cancer, we developed an IL‐22 knockout spontaneous breast cancer mouse model. PyMT mouse model is considered a good model to study the breast tumorigenesis as it closely reflects the biology of human breast cancer. In this model, hyperplasia or increased epithelial cell proliferation occurs at 4–6 weeks of age, adenoma/mammary intraepithelial neoplasia (MIN) or advanced premalignant lesions at 6–8 weeks, early carcinoma or initial stage of malignant transition at 8–10 weeks, and late carcinoma or advanced invasive carcinoma at 10–14 weeks. These mice also develop lung metastasis by the age of 12–14 weeks (Lin *et al.*, 2003). Using these mice, we first investigated whether inhibition of IL‐22 activity can influence the breast tumor development. We interbred the IL‐22^+/+^or IL‐22^−/−^ mice with mice expressing the PyMT oncogene under the control of the MMTV promoter. Cohorts of female mice carrying IL‐22^+/+^or IL‐22^+/−^ or IL‐22^−/−^PyMT genotypic combinations were monitored for mammary tumor development by whole‐mount mammary gland carmine staining. The results of carmine staining analyses of 4‐ to 10‐week‐old mice showed a significant reduction in the growth of breast tumors during malignant transition stage in IL‐22^−/−^/PyMT mice (Fig. 1A). Quantification of the percentage of area occupied by hyperplastic lesions in mammary gland showed significant reduction (*P* < 0.01) in IL‐22^−/−^/PyMT mice compared to control mice at 10 weeks of age (Fig. 1B). However, no difference was observed in tumor formation during early stages of tumor progression between control and IL‐22^−/−^/PyMT mice (6 and 8 weeks; Fig. S2). IL‐22^+/−^/PyMT mice also showed a reduction in the area of hyperplastic lesions compared to control mice (Fig. 1B). Similarly, there was a significant reduction in the total mammary gland weight in IL‐22^−/−^/PyMT mice compared to control mice at 10 weeks of age (*P* < 0.01; Fig. 1C). IL‐22^+/−^/PyMT mice showed no difference (*P* > 0.05) in the mammary gland weight compared to control mice (Fig. 1C). These observations suggest that inactivation of IL‐22 gene inhibits the breast tumor progression. Next, we examined whether the reduced tumor growth at 10 weeks of age in IL‐22^−/−^ mice is a consequence of defective mammary gland development due to IL‐22 gene deletion. Mammary gland development involves extensive epithelial cell proliferation, and inhibition in cell proliferation can slow down the gland development and may contribute to the delay in the overall disease development. Inguinal mammary glands from 5‐ to 10‐week‐old IL‐22^+/+^, IL‐22^+/−^, and IL‐22^−/−^ female mice were used for whole‐mammary gland carmine staining. The results of carmine staining revealed no difference in the ductal growth between mammary glands from IL‐22^+/+^ and IL‐22^−/−^ female mice (Fig. 1D,E). These results show that IL‐22 gene deletion has no effect on normal mammary epithelial cell proliferation and gland development.

### 3.2 IL‐22 gene ablation inhibits malignant transition stage of breast tumor progression

**Figure 2:**
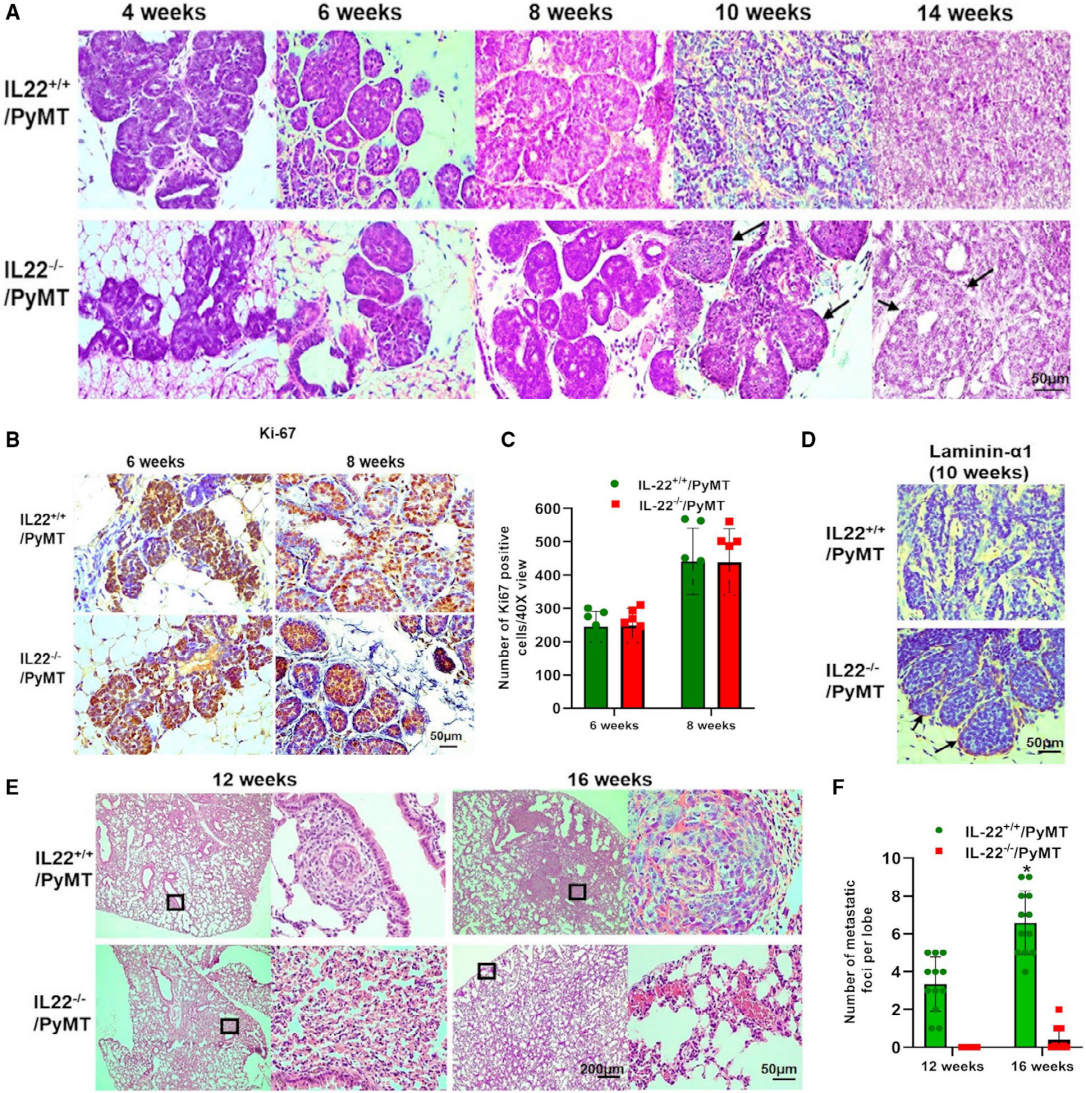
IL‐22^−/−^/PyMT mice show inhibition in the malignant transition stage and reduced lung metastasis of breast cancer. (A) Representative images of hematoxylin and eosin staining of tissue sections prepared from 4‐ to 14‐week‐old virgin IL‐22^+/+^or IL‐22^−/−^PyMT mouse breast tumors. In IL‐22^−/−^PyMT mouse tumor sections, the presence of basement membrane is depicted by black arrows. Magnification 40X; scale bar: 50 µm. The observations were made using *n* = 12 animals in each IL‐22^+/+^ and IL‐22^−/−^PyMT mouse group for each time point. (B) Representative image of Ki‐67 staining of tissue sections prepared from 6‐ and 8‐week‐old virgin IL‐22^+/+^or IL‐22^−/−^PyMT mouse breast tumors. Brown color shows the presence of Ki‐67‐positive cells. Magnification 40X; scale bar: 50 µm. (C) Graph shows quantification of Ki‐67‐positive cells. The total number of Ki‐67‐positive cells per 40X view was counted. A mean of 10 views were considered per sample. Values are presented as mean ± SD, *n* = 7 per group. (D) Representative image of laminin‐1α staining of tissue sections prepared from 10‐week‐old IL‐22^+/+^ (*n* = 10) or IL‐22^−/−^PyMT (*n* = 11) mouse breast tumors. Brown color shows the presence of basement membrane. Magnification 40X; scale bar: 50 µm. (E) Representative images of hematoxylin and eosin staining of tissue sections prepared from 12‐ and 14‐week‐old IL‐22^+/+^or IL‐22^−/−^PyMT mouse lung tissues. Inset boxes shown in the right panel indicate metastatic lesions at higher magnification (10×, scale bar: 200 µm; and 40×, scale bar: 50 µm). (F) Graph shows quantification of the number of metastatic foci per lobe. Values are presented as mean ± SD, *n* = 12 for each time point and each group, *P < 0.01.

To investigate the inhibition in the tumor growth in IL‐22^−/−^/PyMT mice, we performed histopathological examinations of mammary glands from 4‐ to 14‐week‐old mice. Analysis of H&E staining showed that tumors were initiated at the same time (4 weeks of age) in IL‐22^+/+^ or IL‐22^−/−^/PyMT mice (Fig. 2A). Also, comparable tissue pathology was observed at this age in both IL‐22^+/+^ and IL‐22^−/−^/PyMT mice where hyperplastic acinus was filled with epithelial cells (Fig. 2A). Similarly, no difference was observed in hyperplasia (4–6 weeks) and adenoma/MIN or advanced‐premalignant‐stage (6–8 weeks) tissue pathology between IL‐22^+/+^ or IL‐22^−/−^/PyMT mice as primary tumors appeared to be confined by basement membrane with florid epithelial cell proliferation in both group of mice (Fig. 2A). Interestingly, an inhibition in early carcinoma or malignant transition stage (10 weeks) was observed in IL‐22^−/−^/PyMT tumors as epithelial cells were still confined by basement membrane, which is depicted by positive staining of basement membrane marker laminin‐α1 (Fig. 2A,D). In contrast to IL‐22^−/−^/PyMT mice, tumors in control mice progressed to the malignant transition stage and cancer cells appeared pleomorphic with moderate variation in nuclear morphology, size, and shape (Fig. 2A). In addition to increased nuclear pleomorphism, staining of the basement membrane marker laminin‐α1 in this area was negative that confirms the abscence of basement membrane (Fig. 2D). The inhibition in malignant transition stage existed in IL‐22^−/−^/PyMT mice until the 14 weeks of age as acinar structures were still visible in primary tumors compared to solid sheets of cancer cells in control mice (Fig. 2A). The similarity in the tissue pathology during hyperplasia (4–6 weeks) and adenoma/MIN (6–8 weeks) between IL‐22^+/+^ and IL‐22^−/−^/PyMT mice was also confirmed by Ki‐67 cell proliferation marker staining. Similar to histopathological analysis, no difference was observed in the number of Ki‐67‐positive cells in 6‐ and 8‐week‐old IL‐22^+/+^ or IL‐22^−/−^/PyMT mice (Fig. 2B,C). The status of lung metastasis was also examined in these mice as PyMT‐induced tumors frequently metastasize to lungs (Lin *et al.*, 2003). As shown in Fig. 2E,F, all IL‐22^+/+^/PyMT mice developed pulmonary metastases by the age of 12 weeks, whereas no lung metastasis was observed in IL‐22^−/−^/PyMT mice through the age of 16 weeks (*n* = 7 per group). These data show that deletion of IL‐22 gene inhibits the malignant transition stage, and that may have resulted in reduced metastasis in IL‐22^−/−^/PyMT mice.

**Figure 3:**
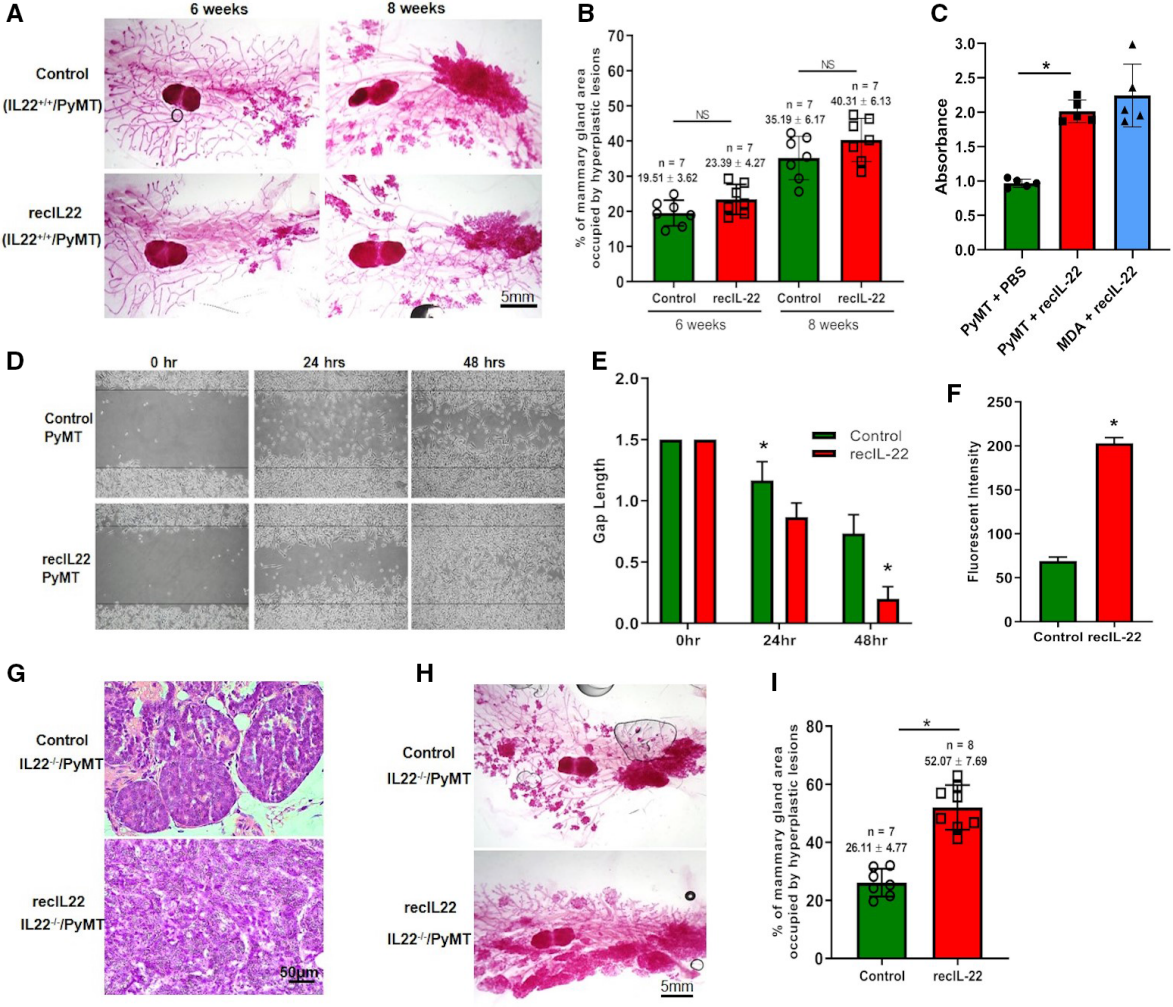
IL‐22 stimulates *in vivo* and *in vitro* cell migration. (A) Representative mammary gland whole mounts from 6‐ and 8‐week‐old virgin IL‐22^+/+^/PyMT mice injected with PBS or recIL‐22 (40 μg·week^−1^; scale bar: 5 mm). (B) Quantification of the area occupied by hyperplastic lesions expressed as a percentage (± SD) of the total mammary gland surface. The number of mice used in the experiment is indicated in the figure. NS, nonsignificant, Student’s *t*‐test. (C) *In vitro* cell proliferation assay: Cancer cells were stimulated with PBS or recIL‐22, and cell proliferation was assessed by MTT assay. Values are presented as mean ± SD; each group of cells was plated and stimulated in triplicate, *n* = 5 individual experiments. *P < 0.01. (D) Wound‐healing assay: Cancer cells were stimulated with PBS or recIL‐22 and used for *in vitro* scratch assay. Images were captured at 0, 24, and 48 h after incubation using a phase‐contrast microscope (40×). (E) Graph shows the migration potential of cancer cells after stimulation with PBS or recIL‐22. Values are presented as mean ± SD; each group of cells was plated and stimulated in triplicate, *n* = 3 individual experiments. *P < 0.01. (F) Tumor‐cell invasion assay: The cancer cells were stimulated with PBS or recIL‐22 and seeded into Matrigel‐coated invasion chambers (24 wells, 8 mm pore size) and allowed to invade toward FBS for 48 h. Invasive cells on the bottom of the membrane were quantified by florescent intensity. Data are reported as mean ± SD, *n* = 5 individual experiments. *P < 0.01. (G) Representative images of hematoxylin and eosin staining of tissue sections prepared from 10‐week‐old virgin IL‐22^−/−^PyMT mouse breast tumors. These mice were injected with PBS or recIL‐22 (40 μg·week^−1^, *n* = 7 per group) from 6 to 9 weeks of age. Magnification 40×; scale bar: 50 µm. (H) Representative mammary gland whole mounts from 10‐week‐old virgin IL‐22^−/−^PyMT mice injected with PBS or recIL‐22 (40 μg·week^−1^; scale bar: 5 mm). (I) Quantification of the area occupied by hyperplastic lesions expressed as a percentage (± SD) of the total mammary gland surface. The number of mice used in the experiment is indicated in the figure. *P < 0.01.

To confirm that IL‐22 has no influence on the cell proliferation during hyperplasia and adenoma/MIN stages of breast tumor progression, i.v. injections of recIL‐22 (40 μg·week^−1^) were given to IL‐22^+/+^/PyMT mice at 4 and 5 weeks of age. The mammary glands from these mice were harvested at 6 (hyperplasia) and 8 (adenoma/MIN) weeks of age and examined for any enhancement in these stages due to elevated levels of IL‐22 in the system. Analysis of whole‐mammary gland carmine staining revealed no difference in the area covered by hyperplastic lesions in recIL‐22‐supplemented IL‐22^+/+^/PyMT mice compared to control IL‐22^+/+^/PyMT mice (*P* > 0.05; Fig. 3A,B) in both the hyperplasia and adenoma/MIN stages. As IL‐22 is known to induce epithelial cell proliferation and a variety of cancer cells proliferate in response to IL‐22 treatment (Lim and Savan, 2014), we next tested our findings *in vitro*. Primary breast tumors were harvested from 8‐week‐old IL‐22^+/+^/PyMT mice. Tumor cells were then purified and cultured in the presence or absence of recIL‐22. Interestingly, in contrast to our *in vivo* findings, these cells responded to recIL‐22 stimulation *in vitro* and showed increased proliferation (2.5‐fold) compared to untreated control cells (Fig. 3C). These results indicate that in this *in vivo* system, breast cancer cells are not dependent on IL‐22 for their proliferation. Next, we investigated whether IL‐22 can enhance migration and invasive behavior of cancer cells *in vitro*. To achieve this, wound‐healing and cell invasion assays were performed on IL‐22^+/+^/PyMT cancer cells treated with recIL‐22 or vehicle control (PBS). Similar to our *in vivo* observations where IL‐22^+/+^/PyMT mice showed increased lung metastasis (Fig. 2E), IL‐22 treatment significantly increased both migration and invasion of cancer cells *in vitro* (*P* < 0.01; Fig. 3D–F). These results confirm that IL‐22 activity is essential for tumor cell malignancy and invasion but not for proliferation in breast cancer.

Next, we tested whether supplementation of IL‐22 can revert the inhibition in the malignant transition stage in IL‐22^−/−^/PyMT mice. Recombinant IL‐22 was injected intravenously (40 µg·week^−1^) in IL‐22^−/−^/PyMT mice from 6 to 9 weeks of age, and tumors were harvested at 10 weeks of age. As shown in Fig. 3G, tumor cells appeared pleomorphic with no sign of basement membrane and disappearance of acinar structures in tumors from recIL‐22‐injected IL‐22^−/−^/PyMT mice, whereas in control IL‐22^−/−^/PyMT mice, tumors appeared to be confined by basement membrane with florid epithelial cell proliferation, which characteristically represents adenoma/MIN stage of tumor progression. In addition, recIL‐22 also increased the overall breast tumor growth in IL‐22^−/−^/PyMT mice as demonstrated by whole‐mammary gland carmine staining (Fig. 3H,I). These results show that IL‐22 is important for malignant transition of breast tumor cells.

### 3.3 IL‐22 is specifically upregulated in tumor microenvironment during the malignant transition stage of breast tumor progression

**Figure 4:**
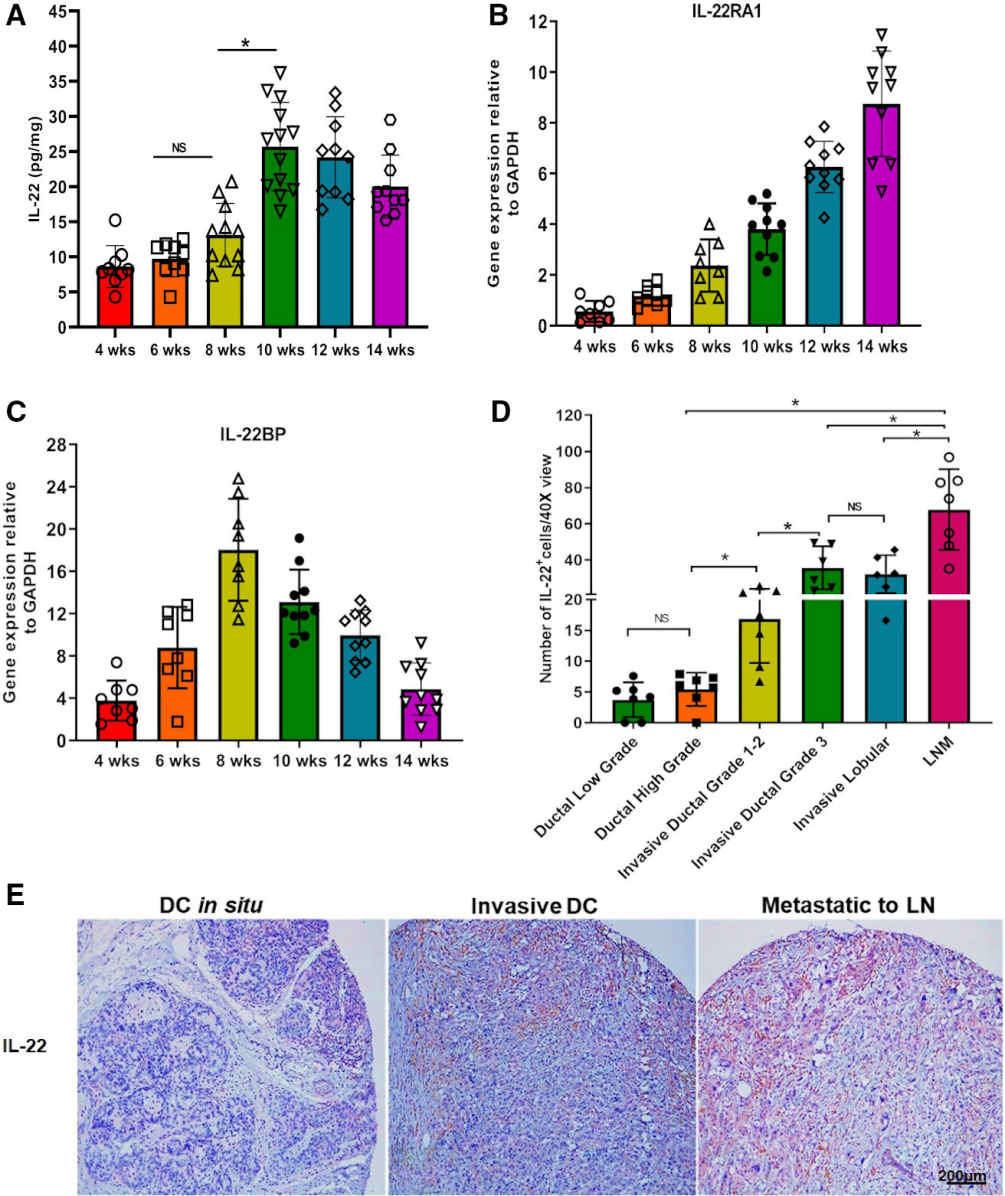
Aggressive breast cancers show increased presence of IL‐22 in TME: (A) Protein levels of IL‐22 in tissues lysates prepared from primary breast tumor tissues from 4‐ to 14‐week‐old IL‐22^+/+^/PyMT mice. IL‐22 protein levels were measured by Luminex assay. Data are reported as mean ± SE, *n* = 9, 9, 11, 13, 10, and 10 mice. *P < 0.01. NS, nonsignificant. (B, C) mRNA levels of IL‐22RA1 and IL‐22BP in primary breast tumor tissues from 4‐ to 14‐week‐old IL‐22^+/+^/PyMT mice quantitated by qPCR. *n* = 8, 8, 8, 10, 10, and 10, *P < 0.01. GAPDH was used as an endogenous control for normalization. The results are presented as mean ± SE. (D) Graph shows quantification of IL‐22‐positive cells in breast tumors from various grade and phenotype breast cancer patients. The total number of IL‐22‐positive cells per 40× view was counted. A mean of 10 views were considered per sample. Values are presented as mean ± SD, *n* = 7, 7, 7, 6, 6, and 7 per group, *P < 0.05. NS, nonsignificant. (E) Representative IHC images showing IL‐22 protein staining in tissues from DC *in situ* and invasive DC, and patients reported with cancer metastatic to LN. Brown color shows positive staining for IL‐22, and blue color shows nuclear staining by the counterstain hematoxylin. *n* = 7; magnification 10×; scale bar: 200 µm.

In breast cancer, elevated levels of IL‐22 are reported in serum and tumor tissues (Rui *et al.*, 2017; Voigt *et al.*, 2017). However, it is not clear whether these elevated levels are associated with a particular stage of cancer progression. We measured IL‐22 protein levels in primary tumors from the initiation, hyperplasia, adenoma, early carcinoma, and late carcinoma stages. Protein expression analysis showed the presence of IL‐22 in TMEs during the initiation and hyperplasia stages (4–6 weeks) of tumor development (Fig. 4A). A significant increase in the levels of IL‐22 was noticed from the early carcinoma stage through the advanced invasive carcinoma stage (8–10 weeks) of disease progression. These levels slightly declined by the end of the late carcinoma stage (14 weeks; Fig. 4A). These results show that IL‐22 is present in the TME from the initiation of the disease; however, its level only increased during the invasion stage, indicating its association with tumor cell malignancy. Additionally, the mRNA expression of IL‐22RA1 and IL‐22BP was also evaluated in primary tumors. As shown in Fig. 4B, the mRNA expression of IL‐22RA1 increased with disease progression. In contrast to IL‐22RA1, IL‐22BP mRNA expression only increased during the initiation and hyperplasia stages (4–8 weeks) and decreased significantly from the early carcinoma stage to the late carcinoma stage (8–14 weeks) of tumor development (Fig. 4C). Next, we investigated if this correlation of IL‐22 with malignancy occurs in human cancer. For this, we used breast carcinoma progression TMA to evaluate IL‐22 levels in different stages of breast cancer progression. This microarray represents breast tumor tissues from low‐/high‐grade DCIS, grade 1–3 invasive DC, LNM‐DC, and invasive lobular carcinoma patients. As shown in Fig. 4D,E and Fig. S3, low‐ and high‐grade DCIS showed no difference in the number of IL‐22‐positive cells. However, the number of IL‐22‐positive cells significantly increased in invasive DC and/or metastatic‐to‐lymph node (LN) DC validating the link between IL‐22 expression and invasiveness of cancer cells. These results strongly show that increased tumor abundance of IL‐22 is associated with malignant behavior of breast cancer cells.

### 3.4 Inactivation of IL‐22 gene affects EMT activation in breast tumor cells

**Figure 5:**
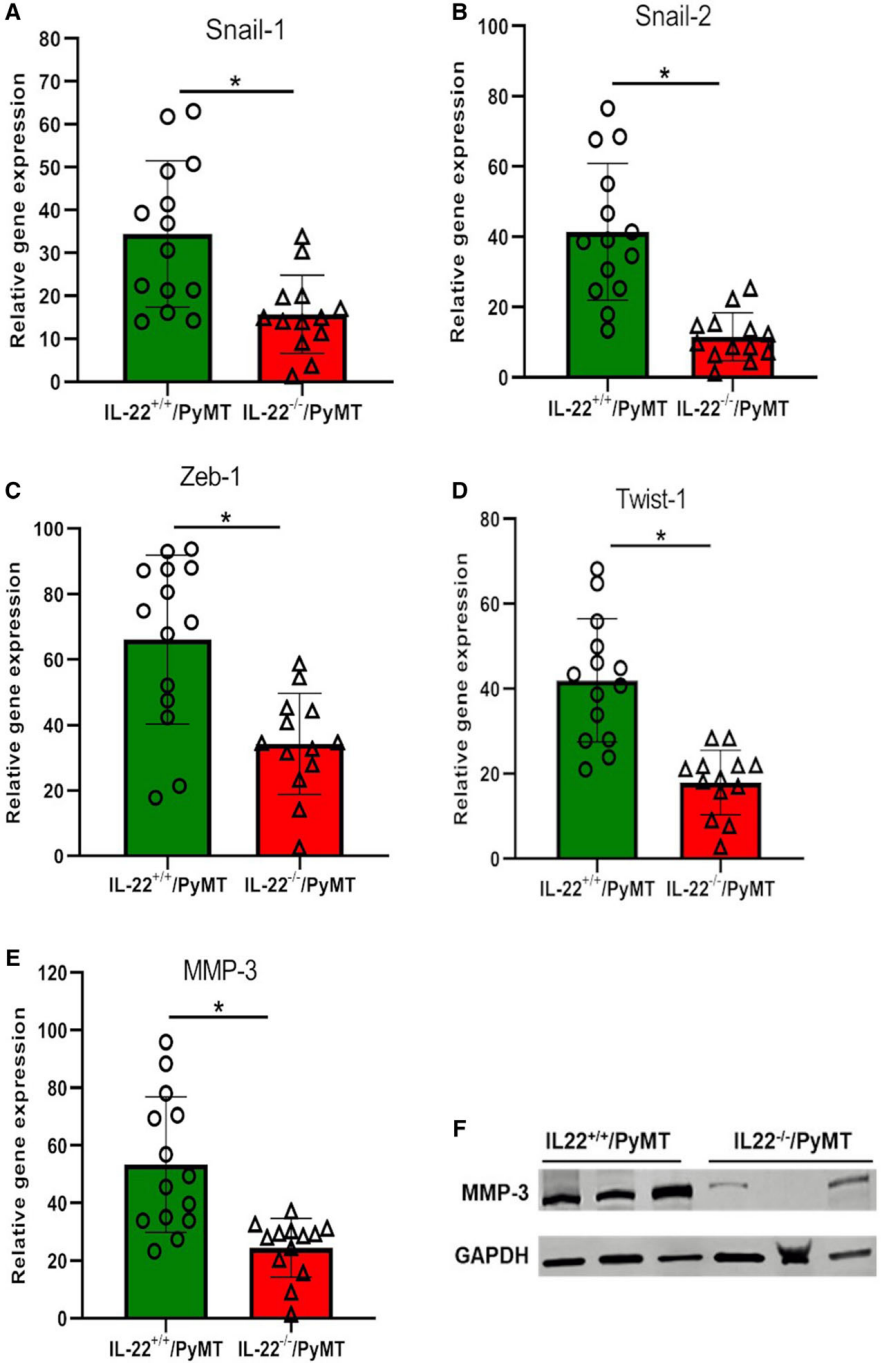
IL‐22^−/−^/PyMT mice show downregulation of the gene expression of EMT‐associated genes: (A–E) mRNA levels of Snail‐1, Snail‐2, Zeb‐1, Twist‐1, and MMP‐3 genes in tumor cells purified from primary breast tumor tissues from 10‐week‐old IL‐22^+/+^or IL‐22^−/−^PyMT mice and quantitated by qPCR. *n* = 14 and 13, *P < 0.01. GAPDH was used as an endogenous control for normalization. The results are presented as mean ± SD. (F) WB showing MMP‐3 protein expression in protein lysates prepared from purified tumor cells from primary breast tumor tissues of 10‐week‐old IL‐22^+/+^or IL‐22^−/−^PyMT mice. *n* = 3 per group. Protein concentrations were normalized using GAPDH.

Epithelial‐to‐mesenchymal transition (EMT) is required by cancer cells for their malignant transformation (Pandya *et al.*, 2017). Various signaling pathways are involved in inducing EMT in cancer including Notch, Wnt, transforming growth factor‐beta, HIF‐1α, and TNF‐α and extracellular matrix stiffness. In response to these pathways, certain EMT transcription factors activate and initiate the EMT process (Yeung and Yang, 2017). To check if IL‐22 deletion affected the EMT in IL‐22^−/−^/PyMT tumors, we analyzed gene expression of EMT‐associated transcription factors Snail‐1, Snail‐2, Twist‐1, and Zeb‐1. Tumor cells of early‐carcinoma‐stage (10 weeks) primary tumors were purified from IL‐22^+/+^ or IL‐22^−/−^/PyMT mice, and mRNA from these cells was used for gene expression analysis. Results showed a significant decrease (*P* < 0.01) in the expression of all transcription factors in tumor cells purified from IL‐22^−/−^/PyMT mice compared to controls (Fig. 5A–D). Gene and protein expression of MMP‐3 was also analyzed in these cells as its expression is known to be upregulated during EMT in breast cancer (Radisky and Radisky, 2010). Both mRNA and protein levels of MMP‐3 were found downregulated in tumor cells from IL‐22^−/−^/PyMT mice compared to control mice (Fig. 5E,F). These results indicate that IL‐22 modulates invasion by regulating EMT in breast cancer cells.

## 4 Discussion

Interleukin‐22 is upregulated in various human cancers, and multiple studies have shown the tumor‐supporting role of IL‐22 in the growth of these cancers (Lim and Savan, 2014). In breast cancer, the role of IL‐22 has been shown in stimulating epithelial cell proliferation, transformation, and migration (Kim *et al.*, 2014; Rui *et al.*, 2017; Voigt *et al.*, 2017; Wang *et al.*, 2018). However, the current understanding of the involvement of IL‐22 in breast cancer tumorigenesis is derived from the use of various human/mouse cancer cell line models. The limitation with these cell line models is that these are transformed advanced‐stage cancer cells and lack the molecular information associated with normal‐to‐cancer cell transformation in epithelial cells. Therefore, these models do not address phenomenon associated with early stages of cancer progression such as hyperplasia and initial stages of malignant transition (early carcinoma and EMT). Here, we showed that inactivation of IL‐22 gene inhibited the malignant transition stage of tumor progression that resulted in an overall inhibition in the growth and metastasis of breast cancer. We also showed that IL‐22 levels substantially increased in the TME during the invasion stage of tumor progression. This correlation of IL‐22 levels with invasion was confirmed in human breast cancer where the number of IL‐22‐positive cells positively correlated with the invasiveness of breast cancer. Additionally, we showed that IL‐22 affects the malignant transition of tumor cells through stimulating EMT in cancer cells.

In breast cancer, it is not established whether elevated levels of IL‐22 are associated with a specific stage of disease progression. Our data showed that IL‐22 is present in the TME from the earliest stages of tumor development, but its expression specifically increased during the early carcinoma/malignant transition stage of tumor progression, suggesting its role in cancer invasion. A recent study has identified T‐helper cell types‐1, ‐17 and ‐22 (Th1, Th17, and Th22) cells as primary source and CD11b^+^ cells as a contributing source of IL‐22 in breast cancer TME (Voigt *et al.*, 2017). Here, the increase in IL‐22 levels during the invasion stage could be due to increased presence of these cells in the TME as in the MMTV‐PyMT breast cancer model, leukocyte infiltration in breast tumor tissues occurs between the early carcinoma and late carcinoma stages (Lin *et al.*, 2003). Our data on human breast cancer where increased number of IL‐22+ cells correlated with invasiveness of cancer also suggest that the presence of IL‐22 in the TME is important for the cancer cell invasion.

Multiple studies have shown that inhibition of IL‐22 activity can hamper cancer cell proliferation and tumor growth (Lim and Savan, 2014; Perusina *et al.*, 2016). In colon cancer, deletion of IL‐22 gene reduced the tumor number as well as overall tumor burden in the intestine (Huber *et al.*, 2012). Similarly, inactivation of IL‐22 gene significantly reduced tumor number and size in lung cancer accompanied by reduced tumor cell proliferation (Khosravi *et al.*, 2018). Our results are in line with these findings as the inactivation of IL‐22 gene reduces both the number of tumor lesions and the overall tumor burden in IL‐22^−/−^PyMT mice. However, this inactivation of IL‐22 gene did not affect the initiation and hyperplasia stages of tumor progression in IL‐22^−/−^PyMT mice. Also, the *in vivo* supplementation of recIL‐22 did not accelerate these stages of tumor progression. In contrast to the *in vivo* system, recIL‐22 effectively stimulated cell proliferation in *in vitro* settings, which is in corroboration with earlier *in vitro* findings on the proliferative effects of IL‐22 on breast cancer cells (Kim *et al.*, 2014; Rui *et al.*, 2017). The discrepancy of cancer cells in response to IL‐22 stimulation in *in vivo* and *in vitro* systems could be due to the presence of IL‐22BP in the TME during these stages. IL‐22BP is a soluble IL‐22 receptor, and it prevents binding of IL‐22 to membranous IL‐22RA1 and blocks downstream signaling by sequestering IL‐22 in the TME. In cancer, increased tumor cell proliferation has been shown upon the inactivation of IL‐22BP in mice (Huber *et al.*, 2012). In our results, IL‐22RA1 expression continuously increased with the tumor progression whereas the expression of IL‐22BP only increased during the cell proliferation stages and declined as the cancer progressed to the invasion stage. This upregulation of IL‐22BP expression during cell proliferation stages may have a neutralizing effect on IL‐22 and prevented its proliferative action on cancer cells. Similarly, the downregulation of IL‐22BP and upregulation of IL‐22 levels during the invasion stage explain the responsiveness of cancer cells to IL‐22.

Interleukin‐22 is also known to induce migration in epithelial cells. It increased the migration of intestinal epithelial cells, promoted invasion and migration of gastric and pancreatic cancer cells, and encouraged metastasis of hepatocellular carcinoma (Brand *et al.*, 2006; Ji *et al.*, 2014; Jiang *et al.*, 2011; Wen *et al.*, 2014). Similarly, IL‐22 has been associated with *in vitro* transformation and migration of breast cancer cells (Wang *et al.*, 2018). The association of IL‐22 with EMT is also reported in keratinocytes and oral squamous cell carcinomas where it suppresses differentiation‐related factors in cells and renders them more susceptible to oncogenic transformation (Lim and Savan, 2014). IL‐22 enhanced the expression of the EMT‐associated transcription factors in primary bronchial epithelial cells from patients with severe asthma (Johnson *et al.*, 2013). Our results are in corroboration with these findings as the stimulation of IL‐22 effectively enhanced the *in vitro* migration and invasion of PyMT cancer cells. The arrest of tumor progression at the malignant transition stage in IL‐22^−/−^PyMT mice suggests its role in invasion and migration. The downregulation of the expression of EMT transcription factors Snail‐1, Snail‐2, Twist‐1, Zeb‐1, and MMP‐3 in IL‐22^−/−^PyMT tumors suggests that IL‐22 mediates invasion through the EMT pathway in breast cancer.

## 5 Conclusion

Our study identifies a previously unrecognized malignant‐stage‐specific role of IL‐22 in breast cancer. We show that (a) IL‐22 is specifically upregulated in the TME during the malignant transition stage of tumor progression; (b) IL‐22 expression correlates with aggressive phenotype in breast cancer; (c) disruption of IL‐22 gene specifically inhibits malignant transition stage; and (d) IL‐22 supplementation reinstates breast cancer malignancy in IL‐22 knockout mice. The study shows that IL‐22 regulates the malignant transformation in cancer cells through EMT pathway that may further affect the invasion in breast cancer.

## Conflict of interest

The authors declare no conflict of interest.

## Author contributions

GKK and AK contributed to designing the research, performing the experiments, interpreting the data, making figures, and writing the paper. SS and VR assisted in animal breeding and experiments for the study. SI assisted in protein assays. KDB edited the paper and provided resources for the study.

## Supporting information


**Fig. S1.** Confirmation of purified tumor cells.
**Fig.** **S2.** Quantification of the hyperplastic lesion area in mammary glands from 6‐ and 8‐week‐old IL‐22^+/+^or IL‐22^+/−^ or IL‐22^−/−^PyMT mice.
**Fig.** **S3.** Representative IHC images showing IL‐22 protein staining in tissues from breast cancer patients.
**Fig.** **S4.** Determination of anti‐IL‐22 antibody specificity.
