# Supplementary material for: Interleukin‐22 promotes development of malignant lesions in a mouse model of spontaneous breast cancer
Source: Mol Oncol. 2019 Dec 4;14(1):211–24. doi: 10.1002/1878-0261.12598 (PMC6944104; doi:10.1002/1878-0261.12598)
Supplement: Supplementary file 1 — Fig. S1. Confirmation of purified tumor cells. Fig. S2. Quantification of the hyperplastic lesion area in mammary glands from 6‐ and 8‐week‐old IL‐22+/+or IL‐22+/− or IL‐22−/−PyMT mice. Fig. S3. Representative IHC images showing IL‐22 protein staining in tissues from breast cancer patients. Fig. S4. Determination of anti‐IL‐22 antibody specificity. [file MOL2-14-211-s001.pdf]

Supplementary Figure 1

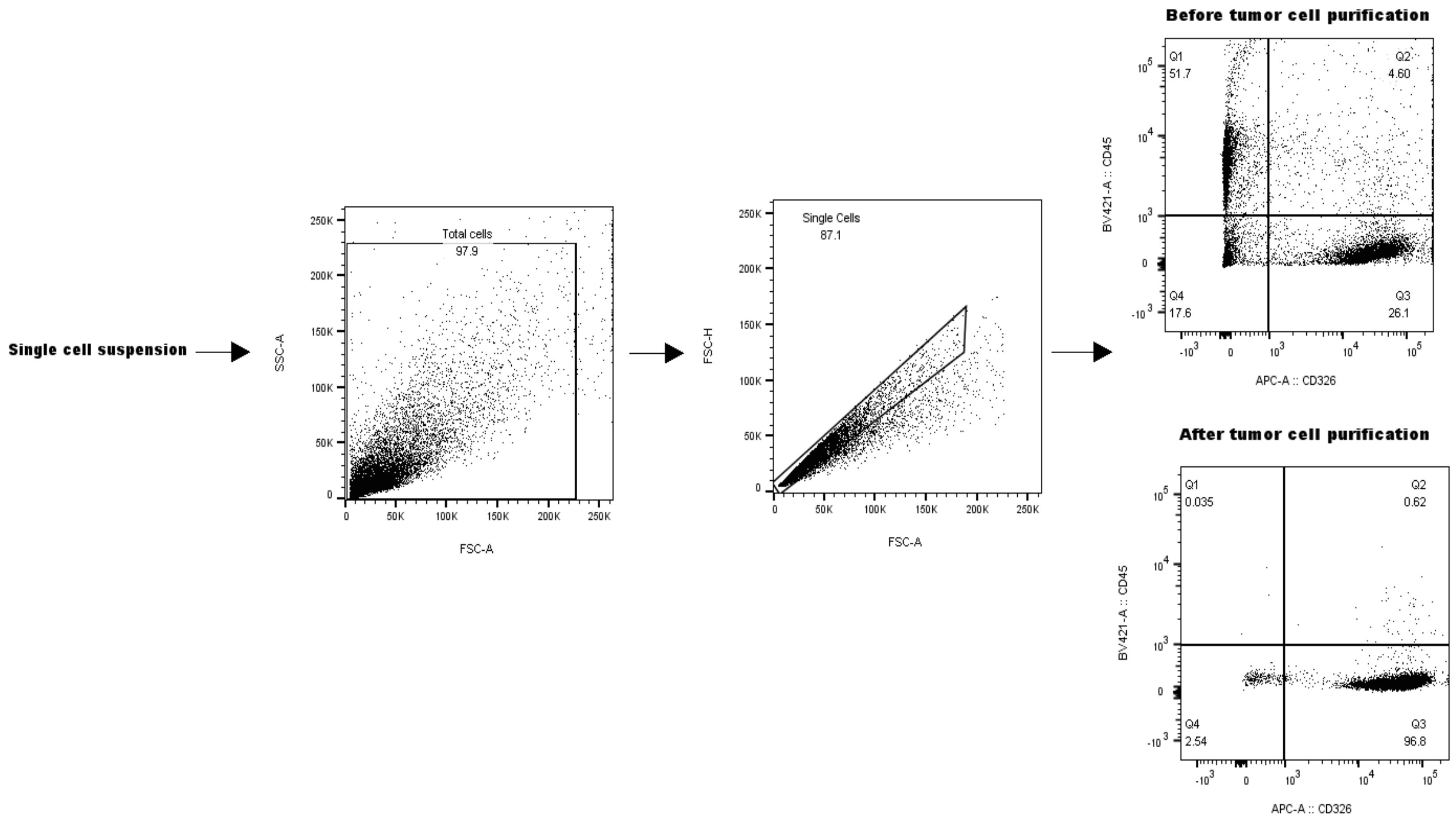

S1. Confirmation of purified tumor cells. Schematic shows the confirmation of tumor cells purified from breast tumors using flow cytometry. Single cell suspensions from tumors were stained with cocktail of antibodies using tumor cell isolation kit (Miltenyi Biotec) and tumor cells were purified through column according to manufacturer's protocol. Purified cells were stained

Supplementary Figure 2

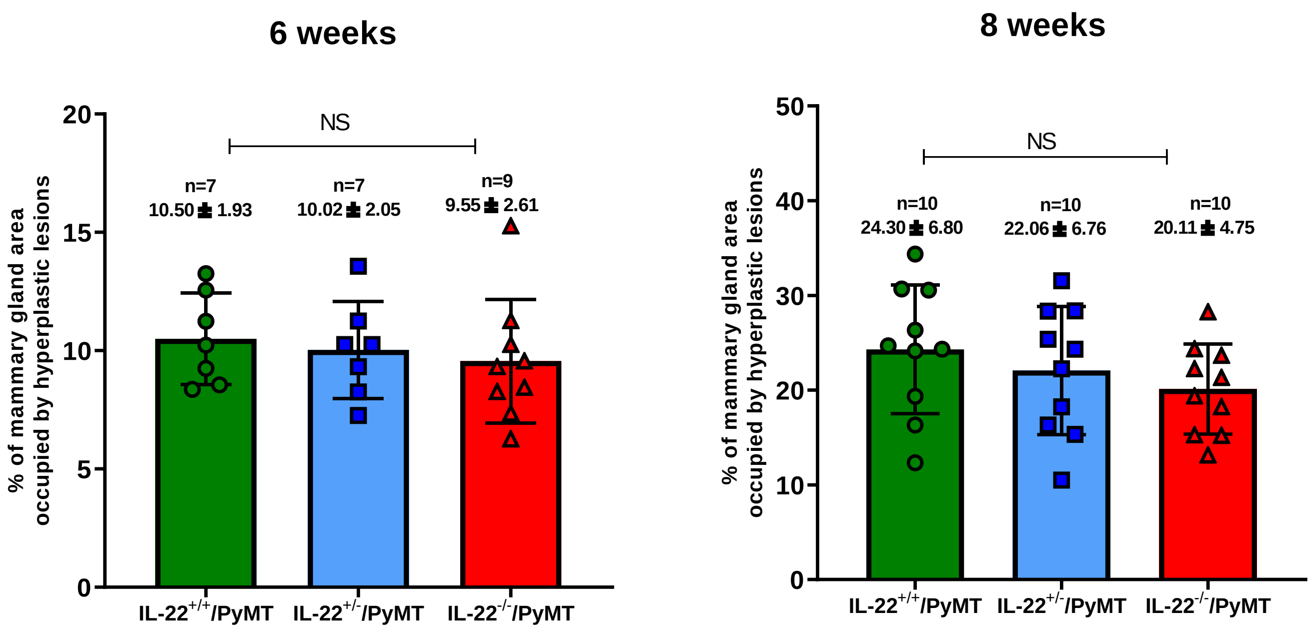

S2. Quantification of the area occupied by hyperplastic lesions expressed as a percentage (±SD) of the total mammary gland surface. Age and number of mice used in the experiment is indicated in the figure. NS, not significant, Student's t test.

Supplementary Figure 3

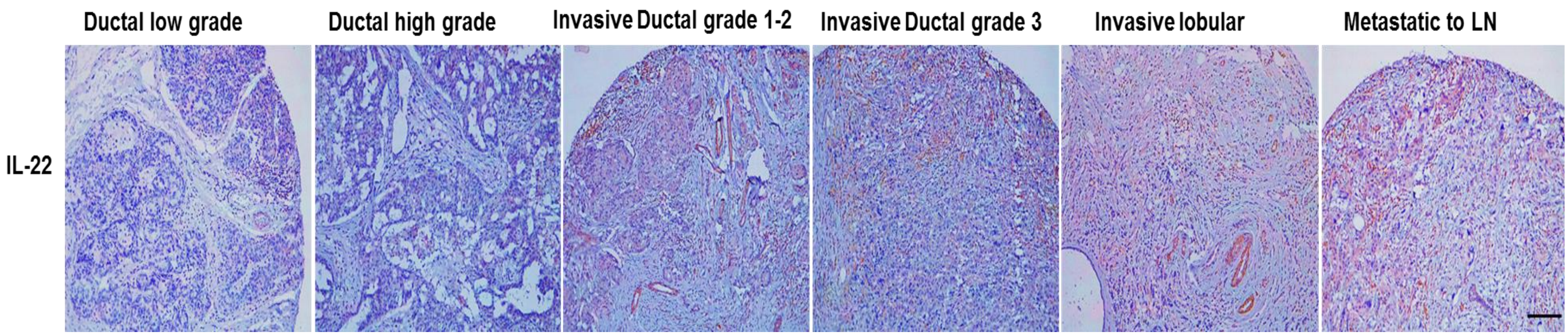

S1: Representative IHC images showing IL-22 protein staining in tissues from ductal carcinoma low grade, high grade, invasive ductal carcinoma grade 1-3, invasive lobular and patients ported with cancer metastatic to lymph node (LN). Brown color shows positive staining for IL-22 and blue color shows nuclear staining by counter stain hematoxylin. n=7, magnification 10X, scale bar 200μm

Supplementary Figure 4

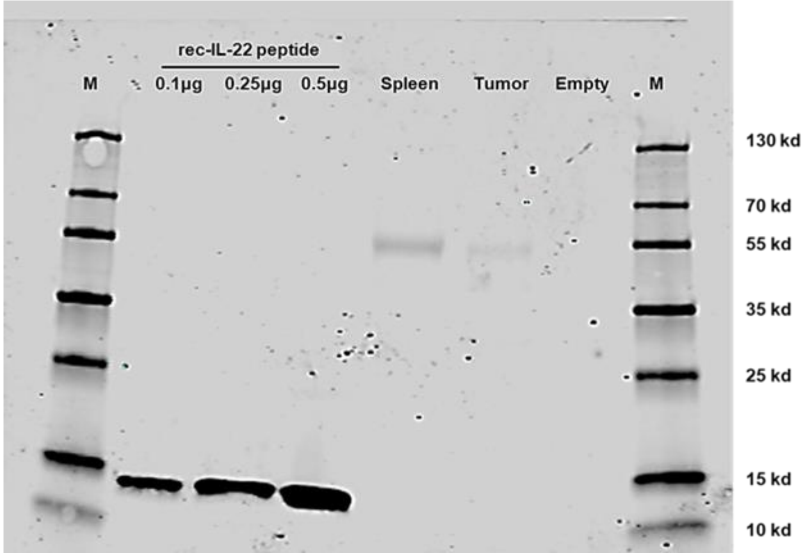

S4. Determination of anti-IL-22 antibody specificity. Western blot detection of IL-22 using anti-IL-22 antibody (Abcam, ab18499) in protein lysates prepared from spleen and breast tumor tissues. recIL-22 peptide was used as positive control. (Total protein 50µg, Anti-IL-22 1:5000 dilution).
